# HELLP! The Novel Use of Continuous Renal Replacement Therapy and Nitric Oxide in the Treatment of Acute Respiratory Failure in HELLP Syndrome

**DOI:** 10.1155/2021/8895319

**Published:** 2021-08-11

**Authors:** Obteene Azimi-Ghomi, Glenn Miller, Carlos Guida, Adrian Marimon, Dessislava Boneva, Mark McKenney

**Affiliations:** Department of Surgery, Kendall Regional Medical Center, 11750 SW 40th Street, Miami, Florida 33175, USA

## Abstract

HELLP (hemolysis, elevated liver enzymes, low platelets) syndrome is a rare but serious complication of pregnancy characterized by hemolytic anemia, elevated liver enzymes, and thrombocytopenia. It occurs in <1% of all pregnancies with 70% of cases developing before delivery, the majority occurring between the 27^th^ and 37^th^ weeks of gestation. Respiratory failure seen in HELLP syndrome clinically and radiographically appears similar to acute respiratory distress syndrome (ARDS), with presence of bilateral pulmonary opacities on imaging as well as persistent hypoxemia requiring elevated ventilator requirements. It is seen to complicate 3-10% of cases of HELLP syndrome. Pulmonary complications are theorized to occur as sequelae of the proinflammatory state induced by HELLP syndrome with endothelial dysfunction and subsequent microangiopathic hemolysis and thrombocytopenia. A robust cytokine inflammatory response similar to ARDS is seen, resulting in noncardiogenic pulmonary edema due to vasoplegia and capillary leak syndrome. We present a case of a 27-year-old uniparous female with a term pregnancy complicated by HELLP syndrome who developed respiratory failure requiring mechanical ventilation. Early CRRT and nitric oxide therapy were initiated, with the patient experiencing clinical and radiological improvement of respiratory function within 48 hours. We document the novel treatment of our patient's acute respiratory failure with CRRT and nitric oxide and delve into the literature regarding its use in acute respiratory failure and ARDS in association with HELLP syndrome.

## 1. Case Report

A 27-year-old G1P0 presented for induction of labor at 40.2 weeks of pregnancy. Due to prolonged fetal deceleration, decision was made to pursue cesarean delivery. Preoperatively, the patient complained of shortness of breath with tachypnea and hypoxemia requiring nasal cannula and subsequently ventimask at 15 L/min of oxygen. The patient was also tachycardic to 120 bpm, with blood pressure 110/68 mmHg. C-section was performed, which was complicated by uterine atony and delayed discovery of placenta accreta. Massive intrapartum hemorrhage occurred that was not amenable to uterine massage, Pitocin, and intrauterine balloon tamponade. Subsequently, an emergent supracervical hysterectomy was performed. The patient afterwards developed diffuse intravascular coagulation and respiratory failure requiring massive transfusion of blood products, intubation, and mechanical ventilation requiring transfer to the surgical intensive care unit. 16 units of packed red blood cells, 12 units of fresh frozen plasma, 2 packs of platelets, and 1 unit of cryoprecipitate were given. The patient was unable to be extubated postoperatively due to elevated oxygen requirements and remained intubated. The patient remained tachycardia with heart rate 130 bpm and blood pressure of 90/64 mmHg.

Postoperative labs demonstrated hemolytic anemia with hemoglobin of 10.7 g/dL with schistocytes and burr cells on peripheral smear and thrombocytopenia with platelets of 37,000. Chemistry and liver panel demonstrated lactic acid of 2.2 mmol/L, AST (aspartate aminotransferase) and ALT (alanine aminotransferase) levels > 700 units/L, total bilirubin of 10.7 mg/dL, and lactic dehydrogenase (LDH) > 3000 units/L. Renal function testing demonstrated blood urea nitrogen and creatinine of 25 mg/dL and 1.2 mg/dL, respectively. The patient required sustained elevated ventilator requirements with persistent hypoxemia despite PEEP (positive end-expiratory pressure) of 20 mmHg and FiO2 (fraction inspired oxygen) of 100%. Arterial blood gases (ABG) demonstrated PaO2/FiO2 ratio of 72 on the above ventilator settings. EKG demonstrated sinus tachycardia. Emergent echocardiography was performed demonstrating preserved ejection fraction of 65%, normal left ventricular diastolic relaxation, left ventricular outflow tract-velocity time integral (LVOT-VTI) of 24 cm, no valvular stenosis/regurgitation, and pulmonary hypertension with RVSP (right ventricular systolic pressure) of 59 mmHg. Chest X-ray demonstrated bilateral diffuse airway opacities suggestive of noncardiogenic edema, with diffuse crackles present bilaterally on chest auscultation. Urine output was approximately 100 cc/hour (patient weight 72.5 kg).

**Figure 1:**
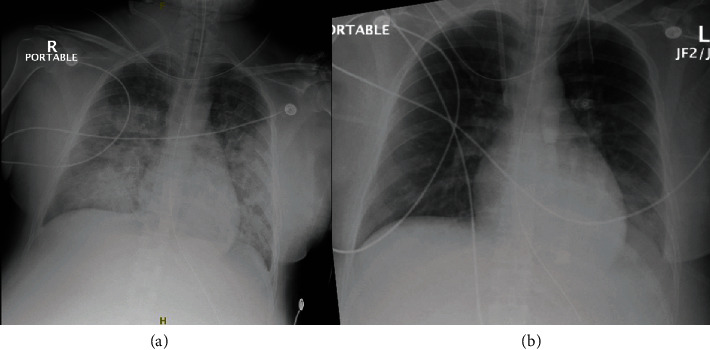
(a) Chest X-ray immediately postoperation demonstrating bilateral diffuse airway opacities demonstrative of acute respiratory distress syndrome. (b) Chest X-ray 72 hours following the index X-ray demonstrating complete resolution of bilateral airway opacities.

The decision was made to start the patient on continuous renal replacement therapy (CRRT) and nitric oxide at 30 ppm. CRRT mode was continuous venovenous hemofiltration (CVVH) at 30 mL/kg/hour. A Baxter AN69 adsorptive hemofilter was utilized. Ultrafiltration only was utilized for the first 16 hours due to patient's hemodynamic instability and then started on 100 cc/h fluid removal. The patient remained on assist-control/volume control settings of 20 breaths/minute at tidal volume of 450 mL with PEEP of 20 mmHg and FiO2 100%. Over the following 24 hours, there was sustained improvement in oxygenation and pO2, with ABG demonstrating PaO2/FiO2 ratio of 213 on PEEP 16/FiO2 80%. Patient's tachycardia and blood pressure improved. The patient was weaned from nitrous oxide 48 hours postoperatively with sustained improvement of arterial O2 content. Repeat echocardiography demonstrated EF of 65%, LVOT-VTI of 19 cm, and improvement in RVSP to 29 mmHg. The patient was weaned to PEEP 8 mmHg and FiO2 50%, with PaO2/FiO2 ratio of 340. The patient's fluid balance was -1100 mL over the 48 hours following initiation of CRRT. The patient was subsequently extubated 72 hours postoperatively to high-flow nasal cannula at 50 L/min at 40% FiO2. Follow-up chest X-rays demonstrated resolution of bilateral airway opacities (Figure 1).

The patient's renal function continued to deteriorate into acute renal failure on hospital day #5 with BUN and Cr of 62 mg/dL and 3.5 mg/dL, respectively, with development of oliguria requiring intermittent hemodialysis for 2 weeks postoperatively. A renal biopsy was performed demonstrating acute tubular necrosis. The patient eventually recovered renal function with improvement of BUN/Cr levels, and hemodialysis was discontinued on hospital day #21. The patient was subsequently discharged on hospital day #23.

## 2. Discussion

HELLP syndrome is a rare but serious complication of pregnancy characterized by hemolytic anemia, elevated liver enzymes, and low platelets (thrombocytopenia) [1]. It occurs in <1% of all pregnancies and in 10-20% of pregnancies complicated by severe preeclampsia [1]. Approximately 70% of cases develop before delivery, with the majority occurring between the 27^th^ and 37^th^ weeks of gestation [1]. The remaining 30% of cases occur within 48 hours of delivery. Patients typically present with abdominal pain, commonly in the right upper quadrant or epigastrium, as well as headache, visual symptoms, and respiratory distress [1, 2]. Hemolysis in HELLP syndrome occurs due to microangiopathic hemolytic anemia as a consequence of the passage of red blood cells through damaged endothelium [1, 2]. Microscopy can demonstrate schistocytes and burr cells. Lactate dehydrogenase levels are typically elevated to >600 units/L. Hemoglobinuria and hyperbilirubinemia are also present. Elevated liver enzymes reflect components of both hemolysis and liver involvement. AST and ALT elevations occur due to liver injury, while hyperbilirubinemia demonstrates both liver injury and hemolysis [1, 2]. Thrombocytopenia occurs due to the activation and subsequent consumption of platelets by the injured vascular endothelium. Developed in 1993 by Martin et al., the Mississippi classification categorizes HELLP syndrome into 3 classes, based on platelet count, transaminase levels (AST and ALT), and lactate dehydrogenase levels (LDH) [3]. Class 1 HELLP syndrome is the most severe, with thrombocytopenia of <50 thousand, elevated AST/ALT levels > 70 units/L, and elevated LDH > 600 units/L [3].

Complications of HELLP syndrome include abruptio placentae, DIC, acute renal failure, ascites, liver hematoma, liver rupture, pulmonary edema, respiratory failure, cerebral hemorrhage, and maternal death [3]. Disseminated intravascular coagulopathy is the most common complication of HELLP syndrome, seen in up to 56% of cases [2, 3]. Pulmonary edema is seen in 3-10% of cases and acute renal failure in 7-35% of cases [2, 3].

Respiratory failure seen in HELLP syndrome clinically and radiographically appears similar to acute respiratory distress syndrome (ARDS), with the presence of bilateral pulmonary opacities on imaging as well as persistent hypoxemia requiring elevated ventilator requirements. It is seen to complicate 3-10% of cases of HELLP syndrome [2]. Pulmonary complications are theorized to occur as sequelae of the proinflammatory state induced by HELLP syndrome with endothelial dysfunction, resulting in subsequent microangiopathic hemolysis and thrombocytopenia [2]. A robust cytokine inflammatory response similar to ARDS is seen with the development of noncardiogenic pulmonary edema due to vasoplegia and capillary leak syndrome [2]. Management of these complications with supportive therapeutic interventions such as noninvasive ventilator support and intubation with mechanical ventilation is typically performed [1, 2]. The use of nitric oxide and CRRT are novel modalities in the management of pulmonary complications of HELLP syndrome. CRRT has been proven to be efficacious in the reduction of circulating cytokines which results in a decreased proinflammatory state [1, 4–6]. This has been shown to decrease vasoplegia and capillary leak syndrome, resulting in a decrease in alveolar fluid accumulation and allowing for improved respiratory function [3–6]. A case report in 2017 by Abascal-Garcia from Mexico demonstrated the utility of early CRRT in the management of respiratory failure in HELLP syndrome. CRRT was initiated following respiratory failure and allowed for weaning of mechanical ventilation and subsequent extubation to occur within 48 hours [6]. CRRT was discontinued 24 hours after extubation, and the patient was subsequently discharged 72 hours after admission [6]. Another report describes the use of CRRT in conjunction with intravenous immunoglobulin (IVIg) in the successful treatment of both pulmonary edema and acute renal failure complicating HELLP syndrome [7]. These cases highlight the potential efficacy of CRRT as the cornerstone treatment for both pulmonary and renal complications of HELLP syndrome.

Nitric oxide is a well-known agent in the treatment of hypoxemia, especially in the neonatal and pediatric population [8]. Nitric oxide is a potent vasodilator via vascular smooth muscle relaxation due to its effect in increasing cyclic guanosine monophosphate (cGMP) that results in selective pulmonary vasodilation when inhaled [8]. Due to its rapid deactivation, systemic effects of nitric oxide are minimal, with studies utilizing inhaled low-dose nitric oxide demonstrating no difference in occurrence of air-leak syndromes (pneumothorax, pneumomediastinum, and pneumopericardium) or creatinine elevations when compared to placebo [9, 10]. Methemoglobinemia is a well-known side effect with inhaled nitric oxide usage, as it oxidizes hemoglobin into methemoglobin and reduces its oxygen-carrying and delivery capacity [11]. Methemoglobin has been shown to occur with low iNO levels (<40 ppm), with the best predictor of the risk of development of methemoglobinemia being cumulative iNO exposure [11]. Nitric oxide has been shown to be effective in the treatment of congenital heart disease, pulmonary hypertension, and cardiac transplantation patients, though its efficacy in the treatment of ARDS has not been clearly established, as numerous trials have failed to demonstrate mortality benefit [8–10]. There have been no reported uses of nitric oxide in the literature in the treatment of respiratory failure in HELLP syndrome. The patient in the case report experienced elevated right heart pressures on echocardiography. The initiation of a multimodal combination of nitric oxide and CRRT helps to quickly reverse the patient's hypoxemia and respiratory failure. This occurred by decreasing right ventricular systolic pressures as well as the systemic proinflammatory cytokine burden, respectively [1, 5, 8]. These measures allowed for weaning of patient's PEEP and O2 requirements. Nitric oxide therapy was started at thirty ppm and weaned to zero over a 48-hour period. This assisted in reducing the patient's right-sided heart pressures from 59 mmHg to 39 mmHg as well as producing radiographic improvement in the patient's pulmonary status as seen by chest X-ray.

## 3. Conclusion

We present a unique case of a uniparous female that developed HELLP syndrome in the peripartum setting complicated by acute respiratory failure requiring mechanical ventilation and elevated FiO2 and PEEP settings. The patient's acute respiratory failure was successfully treated with a multimodal therapy of CRRT and nitric oxide. There has to date been only one documented use of CRRT in the treatment of HELLP syndrome-associated acute respiratory failure. Nitric oxide use has to date never been reported in the use of HELLP syndrome-associated respiratory failure. Acute respiratory failure in the setting of HELLP syndrome presents clinically and radiographically similar in appearance to ARDS, and treatment is supportive, typically requiring mechanical ventilation with high ventilator settings. CRRT and nitric oxide are useful modalities that can allow for decreased time for mechanical ventilation and improved return of pulmonary function, with CRRT demonstrating documented utility in decreasing the cytokine burden and subsequent proinflammatory state. Further investigation is required to assess the broad application of these therapeutic interventions in the future for patients with respiratory failure associated with HELLP syndrome as well as ARDS.

## Data Availability

Data was obtained from below mentioned references as well as patient chart via electronic medical records.
